# Bempedoic Acid’s Use as an Adjunct in Lowering Low-Density Lipoprotein Cholesterol in Patients With Coronary Artery Disease: A Systematic Review

**DOI:** 10.7759/cureus.29891

**Published:** 2022-10-04

**Authors:** Raman Goit, Samia E Saddik, Sarah N Dawood, Ahmad M Rabih, Ahmad Niaj, Aishwarya Raman, Manish Uprety, Maria Jose Calero, Maria Resah B Villanueva, Narges Joshaghani, Nicole Villa, Omar Badla, Safeera Khan

**Affiliations:** 1 Internal Medicine, California Institute of Behavioral Neurosciences & Psychology, Fairfield, USA; 2 Pediatrics, California Institute of Behavioral Neurosciences & Psychology, Fairfield, USA; 3 Obstetrics and Gynecology, California Institute of Behavioral Neurosciences & Psychology, Fairfield, USA; 4 Psychiatry and Behavioral Sciences, California Institute of Behavioral Neurosciences & Psychology, Fairfield, USA; 5 General Surgery, California Institute of Behavioral Neurosciences & Psychology, Fairfield, USA

**Keywords:** arteriosclerotic cardiovascular disease, lipid-lowering drugs, statins, coronary artery disease, bempedoic acid

## Abstract

Coronary artery disease (CAD) is one of the leading causes of death worldwide. Atherosclerosis begins in childhood as fatty streaks, progresses with age, and lifestyle influences the progression of atherosclerotic plaque. Over time, with significant narrowing of the blood vessels, blood flow into the coronary arteries is compromised, resulting in various symptoms of coronary heart disease. Many drugs are used in clinical practice to prevent atherosclerotic cardiovascular events in patients with CAD. This review aims to investigate the efficacy and safety of a non-statin novel lipid-lowering drug, bempedoic acid (BDA), an adenosine triphosphate (ATP) citrate lyase inhibitor, in lowering serum low-density lipoprotein cholesterol (LDL-C) levels among patients with CAD. BDA is a new drug that recently got approval for clinical use. Following its discovery, BDA has been researched in order to investigate its role in the treatment of hypercholesterolemia. A search for studies was conducted using databases such as PubMed, PMC, ScienceDirect, and Google Scholar up until April 30, 2022. This systematic review has followed the Preferred Reporting Items for Systematic Reviews and Meta-Analyses (PRISMA) guidelines.

A total of 11 studies were finalized to explore the role of BDA alone or as an adjunct in lowering serum LDL-C levels in high-risk patients under maximally tolerated statins, statin-intolerant groups, or treatment with other lipid-lowering drugs. These studies are three randomized controlled trials (RCTs), one pre-proof RCT, two systematic reviews and meta-analyses, and five narrative review articles. This review included 8465 participants from recently conducted RCTs and systematic reviews. Another 14014 participants, enrolled for the Cholesterol Lowering via Bempedoic Acid, an Adenosine Triphosphate-Citrate Lyase-Inhibiting Regimen (CLEAR) Outcomes clinical trial, were also included. BDA in combination with ezetimibe showed good evidence of LDL-C lowering effect. Patients on maximally tolerated statin failing to achieve desired LDL-C when treated in combination with BDA showed a significant decrement in serum LDL-C levels, high sensitivity C-reactive protein (HsCRP), and triglyceride. BDA use showed no adverse side effects. The most common side effect seen in several trials was the rise in serum uric acid level. When treating patients with BDA, baseline uric acid levels should be obtained and regular monitoring of uric acid should be done. The CLEAR Outcomes trial, scheduled to be completed by December 2022, will provide further information on BDA. BDA appears to be a promising alternative to currently available secondary lipid-lowering agents.

## Introduction and background

Atherosclerotic cardiovascular disease (ASCVD) is one of the most common causes of premature death worldwide. In clinical practice, it is seen as chronic stable angina, unstable angina, sudden cardiac death, myocardial infarction, and ischemic cardiomyopathy. One hundred and twenty-six million individuals, 1.72% of the world's population, have been affected by coronary artery disease (CAD), with approximately 1655 cases per 100,000 individuals [1]. According to World Health Organization (WHO), in the year 2019, 17.9 million people died of cardiovascular disease (CVD) [2]. It is more prevalent in men than in women. Lifestyle, environment, and genetic factors are strongly related to the development of CAD. The cause of the premature onset of CAD is highly associated with genetics. Genome-wide studies have shown a relationship between premature CAD and chromosome 9p21.3 [3]. The significant risk factors for CAD are increased low-density lipoprotein cholesterol (LDL-C), decreased high-density lipoprotein (HDL) cholesterol, high blood pressure, family history, diabetes, smoking, men above age 45, and post-menopausal women [4].

Atherosclerosis is the pathophysiologic mechanism for ischemic heart disease. Modern advances in managing cardiovascular disease and the introduction of percutaneous coronary intervention and the use of stent have reduced the mortality of this disease. However, lipid-lowering therapy has been the mainstay of cardiovascular risk reduction and prevention, with statins significantly reducing serum cholesterol and stabilizing atherosclerotic plaque [5]. Bempedoic acid (BDA) is a non-statin antihyperlipidemic drug developed by Esperion Therapeutics to treat hypercholesterolemia. It showed promising results in phase III CLEAR clinical trial program; thus, BDA has been used as monotherapy and in combination with ezetimibe in both the United States and European Union [6]. This systematic review attempts to unravel the role of BDA, a non-statin medication, in lowering serum LDL-C levels in treating patients with CAD.

## Review

Methods

Search Strategy

Our research queries followed the Preferred Reporting Items for Systematic Reviews and Meta-Analyses (PRISMA) criteria and principles, and the systematic review was conducted under the respective standards and principles [7].

PubMed, PubMed Central (PMC), ScienceDirect, and Google Scholar were primarily used as major research literature databases, and searches were carried out from April 15, 2022, to April 30, 2022. The searches were conducted using key terms and a medical subject heading (MeSh) and major (Majr) method, which resulted in the findings and discovery of similar papers on the association between BDA and cardiovascular/coronary artery disease. "Bempedoic acid" and "coronary artery disease" were the keywords used in the literature search. The MeSH strategy used in PubMed for the previously used keywords as: Coronary Artery Disease OR Coronary Heart Disease OR Cardiovascular Disease OR (( "Coronary Artery Disease/drug therapy"[Mesh] OR "Coronary Artery Disease/therapy"[Mesh] )) AND ( "Coronary Artery Disease/drug therapy"[Majr] OR "Coronary Artery Disease/therapy"[Majr] ) AND bempedoic acid OR "8-hydroxy-2,2,14,14-tetramethylpentadecanedioic acid" [Supplementary Concept] AND cholesterol OR ( "Cholesterol, LDL/adverse effects"[Majr] OR "Cholesterol, LDL/therapeutic use"[Majr] OR "Cholesterol, LDL/toxicity"[Majr] ). Booleans "AND" and "OR" were used.

The total number of articles selected from PubMed was 123. One hundred and twenty-seven papers were taken from ScienceDirect, nine from PMC, and 42 from Google scholar related to this systematic review. Additional databases, such as Cochrane library and Elton Bryson Stephens Company (EBSCO) did not yield any papers relevant to our queries. Gray literature was omitted from this analysis.

Eligibility Criteria

To explore the role of BDA in treating patients with coronary artery disease (CAD), articles such as randomized controlled trials (RCTs), narrative review articles, systematic reviews, and meta-analyses were collected. These articles compared BDA and other lipid-lowering drugs, efficacy, and side effects. Articles included for the review were selected to meet the following criteria: a) Papers that were published within the last five years; b) full-text papers; c) Patients with pre-existing atherosclerotic cardiovascular disease who are receiving maximally tolerated statins or intolerant to statins, other lipid-lowering agents in addition of BDA alone or in combination to achieve desired reduction of LDL-C; d) Articles available in English language; and e) Articles involving adult population aged between 18 and 85.

Papers published before 2018, articles in other languages, papers containing BDA in treating hyperlipidemia and hypocholesteremia alone, papers discussing pediatric patients, unfinished papers, and books were excluded.

Article Screening and Eligibility Assessment

Titles or abstracts screened articles to disqualify those not relevant to our topic. After that, inclusion and exclusion criteria were used to screen the remaining papers. Then the remaining articles were investigated thoroughly, and a quality assessment was conducted systematically. The articles that passed the quality control standards were finalized to be included in the review. Two independent investigators (RG and SA) did data collection, selection, assessment, and analysis of collected data in each step. We finalized five RCTs, two systematic reviews, and 10 narrative review articles for the quality appraisal test in our review.

Risk Bias in Individual Studies

The selected articles were assessed for quality assessment and risk bias using tools based on study type. The Joanna Briggs Institute (JBI) Critical Appraisal Checklist was used for RCTs; the Assessment of Multiple Systematic Reviews 2 (AMSTAR 2) was used for systematic reviews and meta-analyses, and the Scale for the Assessment of Narrative Review Articles (SANRA) was used for narrative review articles [8-10]. Each assessment tool has its criteria and different scoring. A score of at least 70% for each assessment tool was accepted. These findings are shown below in Table 1. 

**Table 1:** Summary of the accepted articles in this review JBI=Joanna Briggs Institute; AMSTAR 2=Assessment of Multiple Systematic Reviews 2;  SANRA=Scale for the Assessment of Narrative Review Articles;  RCT=randomized controlled trial, PICO=population, intervention, comparison, outcomes; RoB=risk of bias.

Quality assessment tool	Type of research	Items and their characteristics	Total score	Accepted score (>70%)	Accepted studies
JBI critical appraisal checklist for randomized controlled trials [8]	RCTs	(1) Was proper randomization used to assign participants to treatment groups? (2) Was allocation to treatment groups concealed? (3) Were treatment groups similar at the baseline? (4) Were participants blind to treatment assignment? (5) Were those delivering treatment blind to treatment assignment? (6) Were outcomes assessors blind to treatment assignment? (7) Were treatment groups treated identically other than the intervention of interest? (8) Was follow-up complete and if not, were differences between groups in terms of follow-up adequately described and analyzed? (9) Were participants analyzed in the groups to which they were randomized? (10) Were outcomes measured in the same way for treatment groups? (11) Were outcomes measured reliably? (12) Was appropriate statistical analysis used? (13) Was the trial design appropriate, and were any deviations from the standard RCT design (individual randomization, parallel groups) accounted for in the conduct and analysis of the trial?	13	10	Ballantyne et al., 2022 [11]; Ballantyne et al., 2020 [12]; Nicholls et al., 2021 [13]; Goldberg et al., 2019 [14]
SANRA [9]	Narrative reviews articles	Six items are the justification of the article's importance to the readership, statement of concrete aims or formulation of questions, description of the literature search, referencing, scientific reason, and appropriate presentation of data. They were scored as 0, 1, or 2.	12	9	Bardolia et al., 2021 [15]; Claessen et al., 2020 [16]; Feldman et al., 2020 [17]; Kosmas et al., 2019 [18]; Furer et al., 2020 [19]
AMSTAR 2 [10]	A systematic review, meta-analysis	Sixteen items: (1) Did the research questions and inclusion criteria for the review include the components of PICO? (2) Did the report of the review contain an explicit statement that the review methods were established before the conduct of the review, and did the report justify any significant deviations from the protocol? (3) Did the review authors explain their selection of the study designs for inclusion in the review? (4) Did the review authors use a comprehensive literature search strategy? (5) Did the review authors perform study selection in duplicate? (6) Did the review authors perform data extraction in duplicate? (7) Did the review authors provide a list of excluded studies and justify the exclusions? (8) Did the review authors describe the included studies in adequate detail? (9) Did the review authors use a satisfactory technique for assessing the risk of bias (RoB) in individual studies included in the review? (10) Did the review authors report on the sources of funding for the studies included in the review? (11) If meta-analysis was justified, did the review authors use appropriate methods for the statistical combination of results? (12) If a meta-analysis was performed, did the review authors assess the potential impact of RoB in individual studies on the results of the meta-analysis or other evidence synthesis? (13) Did the review authors account for RoB in individual studies when interpreting/ discussing the results of the review? (14) Did the review authors provide a satisfactory explanation for and discussion of any heterogeneity observed in the results of the review? (15) If they performed quantitative synthesis, did the review authors carry out an adequate investigation of publication bias (small study bias) and discuss its likely impact on the results of the review? (16) Did the review authors report any potential sources of conflict of interest, including any funding they received for conducting the review? Scored as YES or NO. Partial Yes was considered as a point.	16	12	Lin et al., 2022 [20]; Wang et al., 2020 [21]

Outcome Assessment

After completing quality assessment studies, patients were grouped according to participants, i.e., patients with cardiovascular disease and a history of treatment with lipid-lowering medications and BDA. Any outcome measure, positive or negative, from the treatment received by the patient was included in the review.

Results

**Figure 1:**
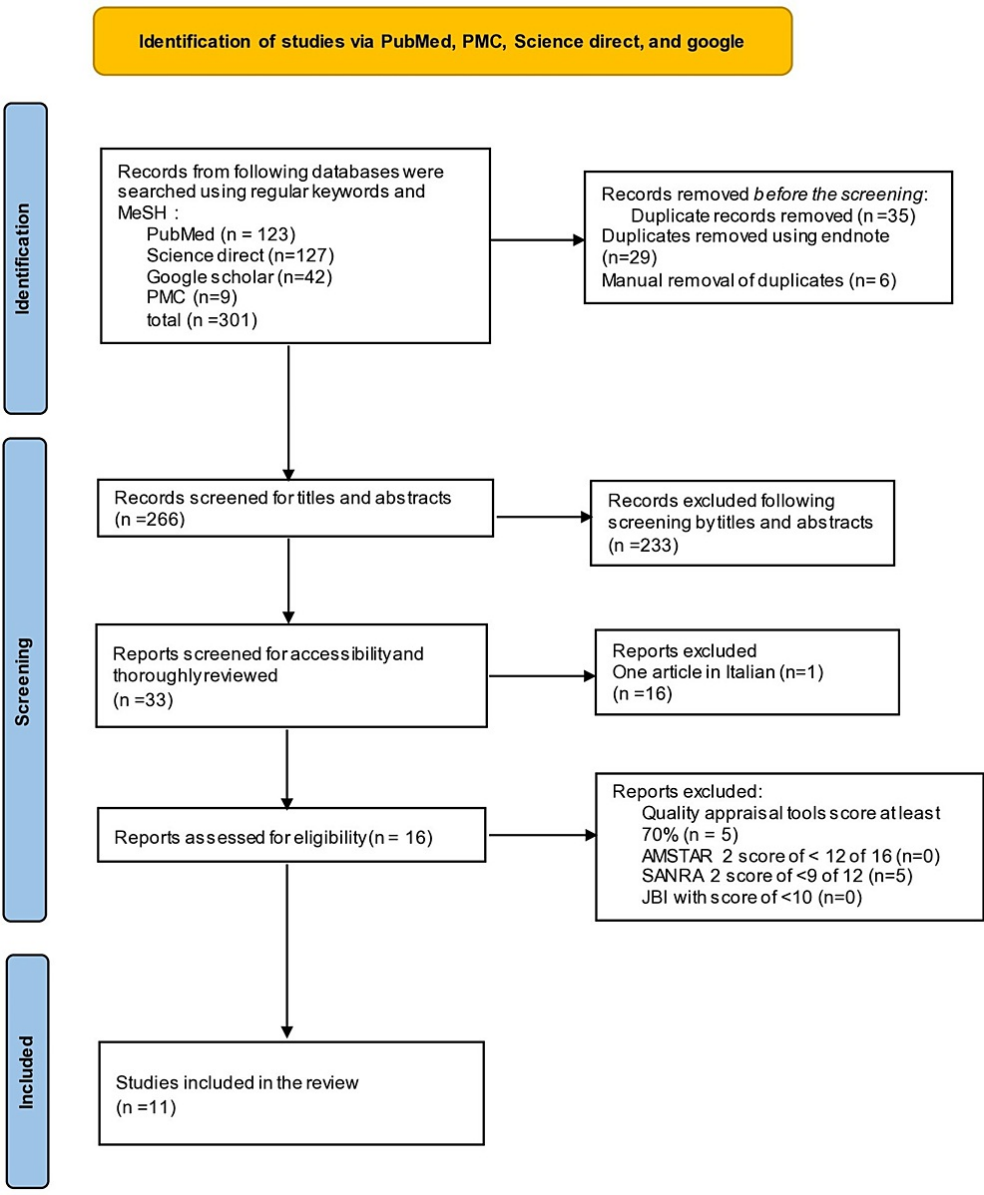
Flow chart of the study search selection. PRISMA=Preferred Reporting Items for Systematic Reviews and Meta-Analyses; JBI=The Joanna Briggs Institute Critical Appraisal Checklist for Randomized Controlled Trials; AMSTAR 2=Assessment of Multiple Systematic Reviews 2; SANRA=Scale for the Assessment of Narrative Review Articles; PMC=PubMed Central; MeSh=Medical Subject Headings

Three hundred and one papers were collected from PubMed, PubMed Central (PMC), Google Scholar, and ScienceDirect databases. Thirty-five duplicate articles were removed using an endnote application and manual screening. The remaining 266 articles were screened by titles, abstracts, or both. Relevant articles were screened using inclusion criteria, and 233 articles that were not relevant were excluded based on exclusion criteria. Finally, 33 remaining articles were thoroughly reviewed. One article was in the Italian language and hence, it was removed. Sixteen articles were excluded based on exclusion criteria. Sixteen articles were finalized for quality appraisal check, which included three randomized control trials, one pre-proof RCT, two systematic reviews and meta-analyses, and 10 review articles. Appropriate quality assessment tools were utilized for the quality appraisal check. Five narrative review articles were removed with a score below 70%. Finally, 11 articles with a score of greater than 70% were accepted in the review. These are three RCTs, one pre-proof RCT, two systematic reviews and meta-analyses, and five narrative reviews. A flow diagram showing the whole screening process and subsequent selection process is depicted in Figure 1. 

RCTs assessed in the review used the JBI critical appraisal checklist for randomized controlled trials. A total of 13 questions analyzed the quality of the RCT, with a total score of 13. To be accepted into the systematic review, a score of 10, i.e., 70%, is required. "Yes," "No," "Unclear," and "NA" were used to answer each question asked. Each "Yes" answer gives one score. Of all four accepted RCTs, two RCTs had item number seven, "Unclear," one had item number eight, "not applicable," and one RCT had item number 13, "Unclear." These items discussed treatment methods, description of adequate follow-up visits, differences between groups in terms of follow-up, and if the review conducted trial design was appropriate. Were any deviations from the standard RCT design (individual randomization, parallel groups) accounted for in the conduct and analysis of the trial? Table 2 shows these findings. 

**Table 2:** Summary of the the JBI critical appraisal checklist for randomized controlled trials by review authors Y=Yes; N=No; U=Unclear; NA=Not Applicable; JBI=Joanna Briggs Institute

First author, year (reference)	Item 1	Item 2	Item 3	Item 4	Item 5	Item 6	Item 7	Item 8	Item 9	Item 10	Item 11	Item 12	Item 13
Ballantyne et al., 2022 [11]	Y	Y	Y	Y	Y	Y	Y	Y	Y	Y	Y	Y	Y
Ballantyne et al., 2020 [12]	Y	Y	Y	Y	Y	Y	U	Y	Y	Y	Y	Y	Y
Nicholls et al., 2021 [13]	Y	Y	Y	Y	Y	Y	Y	NA	Y	Y	Y	Y	Y
Goldberg et al., 2019 [14]	Y	Y	Y	Y	Y	Y	U	Y	Y	Y	Y	Y	U

Two studies were systematic reviews and meta-analyses. These studies were assessed using the AMSTAR 2 tool. One of the accepted reviews had "NO" in items 5, 6, 7, and 16. The other review had "NO" in items 14, 15, and 16. The accepted systematic reviews and meta-analyses had at least a score of "12". These discussed study selection, data extraction from duplicates, justification for excluded studies, and heterogeneity and funding sources, as presented in Table 3. 

**Table 3:** Result summary of critical appraisal for systematic reviews and meta-analyses by review authors Y=Yes; PY=Partial Yes; N=No

First author, year(reference)	Item 1	Item 2	Item 3	Item 4	Item 5	Item 6	Item 7	Item 8	Item 9	Item 10	Item 11	Item 12	Item 13	Item 14	Item 15	Item 16
Lin et al., 2022 [20]	Y	PY	Y	Y	N	N	N	Y	Y	Y	Y	Y	Y	Y	y	N
Wang et al., 2020 [21]	Y	Y	Y	Y	Y	Y	Y	Y	Y	Y	Y	Y	Y	N	N	N

Table 4 demonstrates the scoring of narrative reviews using the SANRA 2 checklist based on six items. For each item asked, scores of 0, 1, and 2 were used. The total score was 12, and five papers scored nine or more out of 12 with 70% or above. For Bardolino et al., Kosmas et al., and Claessen et al., the statement of concrete aims was formulated but not correctly; hence got a score of 1. For Kosmas et al. and Furer et al., the researchers did not explicitly justify the article's importance; hence got a score of 1. For Bardolino et al., Claessen et al., and Feldman et al., search strategies were not presented by the researchers; therefore were scored 0. Kosmas et al. and Furer et al. briefly described search strategies and got a score of one each. Articles Claessen et al. and Furer et al. provided appropriate evidence introduced selectively for scientific reasoning, thus scoring 1. For other criteria, all selected articles got a total score of 2. 

**Table 4:** Result summary of quality assessment of narrative reviews by review authors

Author and year of publication and (reference)	Justification of the article's importance for the leadership	Statement of concrete aims or formulation of questions	Description of the literature search	Referencing	Scientific reasoning	Appropriate presentation of data
Bardolino et al., 2021 [15]	2	1	0	2	2	2
Claessen et al., 2020 [16]	2	2	0	2	1	2
Feldman et al., 2020 [17]	2	1	0	2	2	2
Giglio et al., 2021 [22]	1	0	1	2	1	2
Grassi et al., 2022 [23]	1	0	0	2	1	2
Kosmas et al., 2019 [18]	1	1	1	2	2	2
Furer et al., 2020 [19]	1	2	1	2	1	2
Arsenault et al., 2018 [24]	1	1	0	2	1	2
Musunuru et al., 2021 [25]	1	0	0	2	1	1
Mourikis et al., 2020 [26]	2	1	0	2	1	1

A Description of the Included Studies

The main characteristics of the clinical trials, narrative reviews, systematic reviews, and meta-analyses are shown in Table 5. Included articles had an adult population from 18 to 85 years old with ASCVD, a history of acute myocardial infarction, stable angina, and heterozygous familial hypercholesterolemia (HeFH). One RCT studied the long-term efficacy and safety of BDA, and one pre-proof RCT is an ongoing long-term trial. Two systematic reviews and meta-analyses, which included six RCTs and 11 RCTs with a total of 3956 and 4391 patients, respectively, studied the efficacy and safety of BDA and outcomes. Similarly, five narrative studies described current lipid-lowering agents in high-risk CVD patients, recent evidence on lipid management, new preventive cardiology highlights, and BDA's safety and efficacy among patients with ASCVD groups. 

**Table 5:** A Summary of the findings from selected review articles HeFH=heterozygous familial hypercholesterolemia; OLE=Open-Label Extension; BDA=bempedoic acid; EZE=ezetimibe; FDC=fixed-dose combination; PCSK9 inhibitor=Proprotein convertase subtilisin/Kexin type 9 inhibitor; apoB=Apolipoprotein B-100; ABC=assess risk, anti-inflammatory, aspirin, body weight, blood pressure, cigarette cessation, cholesterol; CLEAR=Cholesterol Lowering via BDA, an adenosine triphosphate-citrate lyase-Inhibiting Regimen; RCT=randomized control trial; CVD=cardiovascular disease; NI=Not indicated; HDL=High density lipoprotein; LDL-C=Low-Density Lipoprotein Cholesterol; HsCRP=High sensitivity C-reactive Protein; ASCVD=Arteriosclerotic cardiovascular disease; ACS=Acute Coronary Syndrome; HsCRP=High sensitivity C-reactive protein

Author, year (reference)	Purpose of the study	Number of patients/ studies	Type of study	conclusion
Ballantyne et al., 2022 [11]	Long-term efficacy as well as safety of BDA in ASCVD and HeFH who completed the CLEAR Harmony parent study	1462	Phase 3 OLE study	As an adjunct with a maximally tolerated statin, BDA is safe and effective for up to two and half years of clinical use in patients with ASCVD and HeFH to treat hypercholesterolemia.
Ballantyne et al., 2020 [12]	BDA 180 mg and ezetimibe 10 mg in a fixed-dose combination compared with placebo, ezetimibe 10 mg alone, and BDA 180 mg alone in patients with hypercholesterolemia at high CVD risk who were receiving maximally tolerated background statin therapy.	382	RCT	Adding BDA + EZE FDC to maximally tolerated statin therapy provides significant atherogenic lipid-lowering compared with either agent alone or placebo.
Nicholls et al., 2021 [13]	In a long-term trial, will adding BDA to standard medical therapy reduce the risk of cardiovascular events in high-risk patients with ASCVD and statin intolerance?	14014	Pre proof for CLEAR Outcomes trial (RCT)	It will be completed by December 2022.
Goldberg et al., 2019 [14]	BDA, combined with ezetimibe or a statin, reduces serum LDL-C, atherogenic lipoproteins, and inflammatory biomarkers in patients with ASCVD, HeFH, or both.	779	RCT	At 12 weeks, significant lowering of LDL-C, non-HDL lipoproteins, total cholesterol, apoB, and HsCRP was reported with side effects like an increased uric acid level in BDA groups.
Lin et al., 2022 [20]	Based on current evidence, investigate BDA efficacy concerning cardiovascular outcomes and BDA safety.	6 RCTS enrolled 3956 patents	Systematic review and meta-analysis	BDA vs. placebo showed no significant effects on primary cardiovascular outcomes in a short-term follow-up study. There was a significant lowering of LDL-C and other atherogenic lipids. Side effects such as muscular disorders, renal function derangement, and gout were observed, and caution was advised.
Wang et al., 2020 [21]	BDA is being studied for its potential benefit and safety in preventing cardiovascular events in high-risk patients.	11 RCTS enrolled 4391 patients	Systematic review and analysis	BDA use in patients with hypercholesterolemia showed a low risk of cardiovascular events and diabetes, significantly decreasing LDL-C levels and HsCRP.
Bardolino et al., 2021 [15]	Current lipid-lowering agents such as ezetimibe, PCSK9 inhibitors, BDA, and inclisiran are used in treating hypercholesterolemia as monotherapy or combination concerning safety, efficacy, and Cardiovascular benefits.	NI	Narrative review	Good safety outcomes were highlighted for novel drugs BDA and inclisiran, and further trials are ongoing to look for additional adverse effects. With current evidence, BDA can be used for high-risk patients, especially those with ASCVD requiring additional LDL-C reduction who are intolerant to statins.
Claessen et al., 2020 [16]	Discussed current evidence on lipid management, early escalation of lipid-lowering therapy, and novel lipid-lowering drugs under investigation to lower cardiovascular events in high-risk patients.	(14014 ongoing trial 4236 meta-analysis of 7 studies) BDA	Narrative review	BDA approval for hypercholesterolemia or established cardiovascular disease with LDL-C > 70 using the most tolerable statin. Specific data on the impact of BDA on LDL-C reduction and outcomes following recent ACS is lacking.
Feldman et al., 2020 [17]	New highlights in preventive cardiology from the 2019 cardiovascular disease guideline in lowering atherogenic lipids and the role of current therapeutic options	NI	Narrative review	The ABC strategy for preventing and reversing genetically influenced ASCVD, as well as the role of aggressive lifestyle and pharmacologic therapy in preventing cardiovascular events
Kosmas et al., 2019 [18]	To discuss current lipid-modifying therapies for the treatment and prevention of CVD, with an eye toward future benefits among high-risk groups.	NI	Editorial review	Novel pharmacological agents significantly lower LDL-C and other atherogenic lipids with good safety outcomes in trials. However, more trials are needed for future approval of these drugs for lowering coronary artery disease.
Furer et al., 2020 [19]	To evaluate the efficacy and safety of BDA in phase three studies and its role in the management of hypercholesterolemia	4050	Narrative review	BDA is an additional non-statin option for patients with hypercholesterolemia or established ASCVD. The outcome of the trial will put more light on the safety aspect.

From our perspective, this is the third systematic review conducted to explore BDA's role in lowering serum low-density lipoprotein cholesterol (LDL-C) in adult patients with CAD, using a pool of data from recent RCTs, narrative review articles and systematic reviews, and meta-analyses altogether. 

A total of three recent RCTs and one pre-proof RCT were included in our review. These studies were conducted on adult patients aged 18 and 85 years old with a history of ASCVD, a history of acute myocardial infarction, stable angina, HeFH, unstable angina, those who were treated by coronary revascularization procedures, cases of coronary artery blockage of ≥ 50% in major vessels diagnosed using invasive procedures or CT angiography, peripheral arterial disease presenting with claudication with evidence on angiogram showing ≥ 50% stenosis or cerebrovascular atherosclerotic disease, which includes ischemic stroke, carotid endarterectomy, stent placement, and 70% blockade on imaging of respective vessel [11-13]. In patients with diabetes, additional risk factors such as men aged 45 and above and females aged 55 and above; family history of premature cardiovascular disease (CVD); smoking history; hypertension; coronary artery calcium score above 95th percentile; low high-density lipoprotein (HDL) and serum LDL-C of more than 100mg/dl on maximally tolerated statin or lipid-lowering drugs were included [12-14].

Two systematic reviews and a meta-analysis were included. Lin et al. included six studies with 3956 patients. Five studies were phase three RCTs, and one was a phase 2b RCT. Three trials were on patients on maximally tolerated statin therapy; three trials were on statin-intolerant groups or those who stopped lipid-lowering therapy [20]. Wang et al. included eleven trials with a total of 4391 participants. This study analyzed significant adverse cardiac events in a population with a history of statin intolerance: participants who took combination pills of BDA and ezetimibe and statin therapy, participants on BDA plus maximally tolerated statin therapy, and changes in LDL-C levels in all groups [21].

Bardolino et al. described pharmacokinetic profiles, drug efficacy, and safety as a monotherapy or in combination in treating patients with hypercholesterolemia. The secondary lipid-lowering drugs included, ezetimibe, Proprotein convertase subtilisin/Kexin type 9 (PCSK9) inhibitors, and new drugs, like BDA and inclisiran [15]. Data for BDA was backed by five clinical trials: CLEAR harmony, wisdom, serenity, tranquility, and outcome trials (ongoing, expected to be released in 2022) [15]. Claessen et al. describe the current state of evidence on lipid management in patients presenting with acute coronary syndrome (ACS). It also benefits early lipid-lowering therapy and novel lipid-lowering drugs currently under trial. It describes a review of the CLEAR and CLEAR wisdom phase three trials and a meta-analysis of seven studies with 4,236 participants to look for the safety profile of novel BDA [16].

Feldman et al. discussed preventive measures taken from the 2019 cardiovascular disease guideline and the European society's advice for preventive strategies for delaying disease outcomes among those who suggested earlier ASCVD pathogenesis. It includes the American college of cardiology (ACC)/the American heart association (AHA) guidelines for risk assessment and 10-year and lifetime risk for ASCVD, and information on coronary artery calcium (CAC) incorporated into the 2019 ACC/AHA prevention guidelines and the 2019 European society of cardiology (ESC)/ the European association for the study of diabetes (EASD) guidelines. Along with these, it highlights the role of BDA in maximally tolerated statin therapy and evidence of LDL-C reduction by 15 to 17% and a reduction of high-sensitivity C-reactive protein (HsCRP), which is backed up by evidence from two studies published in 2019: the CLEAR harmony and CLEAR wisdom trials [17]. The Kosmas et al. editorial review, published in the World Journal of Cardiology, discusses the current clinical and scientific data on new and promising lipid-modifying therapies in CVD management. It also includes studies from one phase 2a clinical trial and the CLEAR wisdom trial, published in the American College of Cardiology 2019 scientific section [18]. Furer et al. reviewed five significant phase three trials named CLEAR (harmony, wisdom, serenity, tranquility, and outcome), out of which four trials were completed. One, which is still ongoing, is investigating the impact of BDA on cardiovascular clinical endpoints. Of these, two trials (harmony and wisdom) studied the use of BDA in patients on maximally tolerated statins. In two trials, namely serenity and tranquility, patients were treated with an oral combination of BDA and ezetimibe in statin-intolerant patients and its role was determined in hypercholesterolemia management [19].

Discussion

CAD is a progressive disease that generally starts in early adolescence and progresses throughout life. Serum cholesterol and LDL-C have a strong relationship with plaque formation and subsequently narrowing blood vessels, leading to cardiovascular disease, which later manifests as myocardial infarction in the heart, peripheral artery disease in the extremities, and stroke in the case of the brain. Several recent guidelines recommend lowering serum LDL-C levels to a certain level for secondary prevention and preventing future cardiovascular events in high-risk patients. Current guidelines state that statin is the mainstay treatment option for lowering serum LDL-C levels. However, the addition of a second or third-line drug has been required to achieve the optimum desired serum LDL-C in high-risk patients.

This systematic review includes 11 articles that have been studied with 8465 participants, and another 14014 patients are still being studied for the CLEAR Outcome trial, estimated to be completed by December 2022. BDA, which is a non-statin novel lipid-lowering drug which can be taken once a day orally, was investigated in this review. The effect of BDA on serum LDL-C levels in patients with a high risk of CVD or established coronary artery disease who are currently managed with maximally tolerated statins alone or statin plus ezetimibe was studied. The side effects of BDA in the patients mentioned above are also thoroughly reviewed in all included articles, and the final findings are discussed appropriately.

BDA's Role in Lowering LDL-C in CAD

BDA comes in a once-daily tablet in 180mg form or in combination with ezetimibe [15,19]. The BDA was approved by the FDA in 2020 [19]. It provides patients with HeFH or established ASCVD an additional non-statin therapy option to lower LDL-C levels [15]. Five large clinical trials have investigated the role of BDA in lowering LDL-C. Four trials are completed, and one CLEAR Outcomes trial is estimated to be completed by December 2022. Currently, over 14014 patients are enrolled in the CLEAR Outcomes study, which is still ongoing. Findings from the CLEAR harmony open-label extension (OLE) study, which suggests good safety and efficacy of BDA in patients with ASCVD and high-risk CVD after 130 weeks of trial, showed overall good outcomes [11-14]. The OLE study showed a change in serum LDL-C levels compared to the parent study in the BDA treatment group, with a mean percentage of change of 14.2 0.9% (16.0 1.0 mg/dl), from baseline to 78 weeks [11]. For the placebo group, following the parent study, there was a similar change in serum LDL-C following the initiation of open-label BDA treatment (-14.5 1.0% [-15.4 1.0mg/dl]) [11]. The CLEAR harmony and wisdom trial, which was done on patients already on maximum therapy with statins, showed a significant reduction in serum LDL-C levels, HsCRP, and triglyceride (TG) [12,20,21]. One phase three trial, BDA in combination with ezetimibe fixed-dose, showed a significant lowering effect on serum LDL-C levels (a reduction of 38%), i.e., a significant proportion of patients had achieved serum LDL-C less than 100 mg/dl (2.6 mmol/L) at 12 weeks of therapy compared to placebo, ezetimibe, and BDA alone [12]. The combination pill also reduced HsCRP by 35.1%, non-HDL cholesterol, total cholesterol, and apolipoprotein B more than placebo, ezetimibe, or BDA. [12]. Two systematic reviews and meta-analyses conducted on six and eleven RCTs found significant benefits of BDA over placebo for non-coronary revascularization patient groups [20,21]. Overall, Wang et al., which conducted a systematic review in more recent times with a more significant compilation of RCTs with nine trials, supported the use of BDA in lowering LDL-C levels in patients with ASCVD and CAD with patients with hypercholesterolemia [21]. Several reviews on BDA conducted in the last five years have shown that taking 180mg of BDA once a day and maximally tolerated statin results in an additional 15% to 20% reduction in serum LDL-C levels. A combination of ezetimibe and BDA could lower serum LDL-C by 35% to 40%. In addition, it has shown a lowering effect of BDA on HsCRP [16,17].

Mechanism of Action and Safety Outcome of BDA

**Figure 2:**
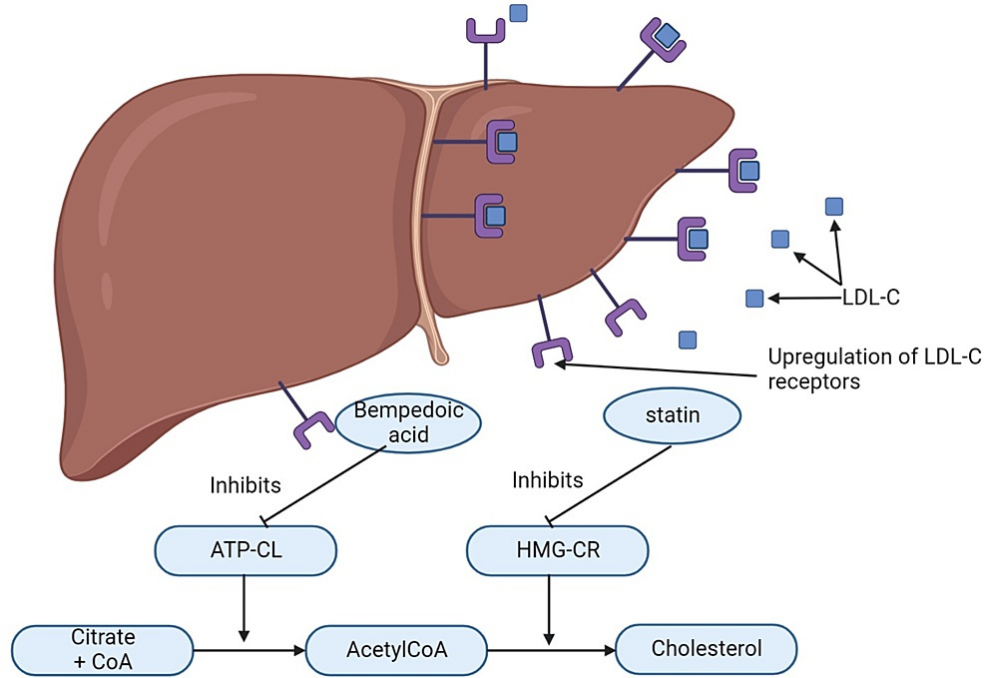
Mechanism of action of bempedoic acid and statin in hepatocytes LDL-C=Low-density lipoprotein Cholesterol; ATP-CL=Adenosine triphosphate citrate lyase; HMG-CR=Hydroxymethylglutaryl-coenzyme A reductase inhibitor; CoA=coenzyme A Image Credit: Author Raman Goit; Created with BioRender.com (BioRender, Toronto, Ontario, Canada)

BDA is a prodrug activated by the enzyme very-long-chain acyl-CoA synthetase A, mainly found in the liver and kidneys [15]. The active metabolite of BDA, i.e., ESP15228, inhibits adenosine triphosphate (ATP) citrate lyase, which also helps produce acetyl CoA from citrate, preventing de novo cholesterol synthesis in the liver. This enzyme is the initial step in cholesterol synthesis only before hydroxymethylglutaryl-coenzyme A (HMG-CoA) reductase [15]. The mechanism of action of BDA and statin is illustrated in Figure 2. After oral intake following first-pass metabolism, the volume of distribution for BDA is 18L [15]. It is highly plasma protein bound and reaches its maximum plasma concentration in 3.5 hours [15]. Drug-drug interactions (DDIs) involved in BDA co-administration with simvastatin and pravastatin, which result in increased statin concertation. The mechanism is unknown, but evidence points to a glucuronide metabolite that weakly inhibits organic anion transporting polypeptide 1B1 (OATP1B1) at a higher dose, i.e., 240mg. This inhibition of OATP1B1 increases plasma concentrations of statins, causing an increased risk for statin-related myopathies and reducing statin efficacy because functional OATP1B1 transporters are required for statin transport into hepatocytes [15]. Administration of simvastatin 40 mg with BDA 180 mg and pravastatin 40 mg with BDA 240 mg resulted in a 96% vs. 99% increase in simvastatin vs. pravastatin area under the curve (AUC) [15]. The increase in the AUC seen for atorvastatin and rosuvastatin was within the normal statin exposure range. BDA with simvastatin doses of > 20 mg or pravastatin doses of > 40 mg is not recommended, and dosage adjustments are not required with atorvastatin or rosuvastatin [15]. BDA has a few drug-disease interactions that require attention. BDA use has been associated with decrease in hemoglobin levels, leukocyte counts, and increase in platelet counts [15]. Several BDA trials reported nasopharyngitis, urinary tract infection, and hyperuricemia. Ballantyne et al. showed increased serum uric acid level and constipation, fatigue, muscle spasm, and oral discomfort in participants in phase three of the trial [12]. One open-label comprehensive study was carried out for over 130 weeks to assess the long-term safety and efficacy of 180mg BDA therapy [11]. The study primarily focused on treatment-emergent adverse events (TEAEs), severe TEAEs, and adverse events of particular interest (AES). The study findings suggested common TEAEs as muscle spasms, myalgia, pain in extremities, arthralgias, and dizziness. There were some reported cases of tendon rupture, but all patients with tendon rupture had a history of tendon injury, statin drug therapy, and advanced age. However, the investigators also stated that none of the cases were related to BDA use. [11]. There were reported cases of gout, the elevation of the creatine kinase enzyme, hepatic enzymes, hypoglycemia, and neurocognitive disorders. This study also showed the effects of BDA on hemoglobin, leucocytes, and platelets, but these findings were non-significant [11]. As a result, patients with such histories should be ruled out before initiating BDA, and caution should be implemented [15].

Two systematic reviews and meta-analyses were performed on the clinical efficacy and safety of BDA. One focused on preventing cardiovascular events and diabetes, and the other focused on outcomes of BDA for LDL-C lowering therapy in patients at high cardiovascular risk [20,21]. Lin et al. favored BDA with low rates of new-onset or worsening diabetes [20,21], but with some side effects such as gout, high serum uric acid levels, muscular disorders, rare cases of worsening GFR, increase in serum creatinine, upper respiratory tract infections, urinary tract infections, and neurocognitive disorders [20]. The positive effect on diabetes was related to the role of BDA in the activation of the adenosine monophosphate-activated protein kinase (AMPK) pathway and, similarly, the inhibition of adenosine triphosphate-citrate lyase (ACL) in the hepatocytes [21]. Wang et al. compared previous systematic reviews and meta-analyses pooled in 11 RCTs until March 3, 2020, and showed a good outcome for BDA. There was a reduction in the composite cardiovascular outcome, a reduction in serum LDL-C levels and CRP levels. In addition, there was evidence of a reduction in rates of new-onset or worsening diabetes [21]. Four studies showed a reduction in new-onset or worsening diabetes in BDA vs. placebo, similar to Lin et al. The serum uric acid level in BDA users was elevated, but glomerular filtration rate (GFR) was unaffected. This review showed no adverse events, serious side effects, or muscle-related adverse events. It also showed no evidence of increased serum creatinine and no incidence of gout, neurocognitive disorders, deranged liver enzymes, or creatine kinase, respectively [21].

BDA vs. Other Non-Statin Lipid-Lowering Agents

Since statins have been the mainstay of lipid-lowering treatment since their approval in 1987 and have remained the gold standard, many patients do not achieve desired LDL-C levels [15]. In the early 2000s, ezetimibe was approved for hypercholesterolemia and then PCSK9 inhibitors with a well-documented safety and efficacy profile [15,16]. New guidelines as of 2020 by ACC/AHA advise the use of PCSK9 inhibitors and ezetimibe as secondary adjunct drugs for lipid management [27]. Inclisiran is another lipid-lowering drug with BDA, but the FDA declined it in 2020 because of an inability to perform a facility inspection [15]. Currently, statins are favored as first-line therapy for the management of dyslipidemia. It has side effects like myalgia, elevated liver enzymes (occurring in 0.5%-3.0% of patients) which indicates less severe hepatic injuries. PCSK9 inhibitors are injectable drugs which have minor side effects like bruising, erythema, or pain [16]. Some evidence of neurocognitive impairments was seen with PCSK9 inhibitors, and are under investigation for possible neurocognitive impairments. Initially, two studies, EBBINGHAUS and FOURIER, which were 19-month studies, showed no difference in cognitive functions. However, a long-term assessment is being planned [16]. BDA, which has similar outcomes but is administered orally, is a good alternative for secondary prevention with good adherence; however, long-term outcomes are still under evaluation [16]. In combination with ezetimibe, BDA has significantly reduced serum LDL-C by 35% to 40% in patients taking maximally tolerated statins [16,17]. At 12 weeks of therapy, BDA in combination with ezetimibe fixed-dose showed a significant lowering effect on serum LDL-C levels (a reduction of 38%), i.e., a significant proportion of patients had achieved serum LDL-C levels of less than 100 mg/dl (2.6 mmol/L) at 12 weeks of therapy [12]. When taken alone and with a maximally tolerated statin, BDA results in an additional 15% to 20% reduction in serum LDL-C levels [16,17]. In our time, BDA could be the best available alternative adjunct option to treat patients with HeFH or with established ASCVD [11]. After completing the CLEAR Outcome trial, more trials must be done on BDA's long-term efficacy and safety. BDA shows to be a promising drug in lowering serum LDL-C levels in patients requiring additional alternative drug therapy to reach desired LDL-C levels.

Limitations

Articles included in this review were recently published, and the risk of bias was assessed by two group members using appropriate quality appraisal tools. The most important strength of our review is that we have collected important information from various articles to support our findings.

Our study limited the included articles to the English language published in four databases from 2018 to 2022. Grey literature and other databases were also not included. Some good clinical trials were not accessible to us. Having few long-duration clinical trials on BDA, there is inconsistency in our conclusion. Therefore, this review recommends more RCTs and observational studies to be conducted with larger sample sizes and longer duration of follow-ups either with BDA alone or in combination in established ASCVD patient groups who are intolerant to statin or didn’t achieve adequate LDL-C levels despite maximum tolerated statin use. In our systematic review one study is still not completed; future studies could benefit greatly. 

## Conclusions

CAD is a fatal disease that can be prevented with proper diet and lifestyle modification. Many clinicians face challenges despite adequate measures taken to lower the main culprit for atherosclerosis formation, i.e., LDL-C, which remains high in some patients despite being on maximally tolerated statin regimens. This systematic review was conducted on 11 articles to find new evidence on the role of BDA as secondary drug therapy for patients who are on maximally tolerated statins or statin intolerant. Although at the moment, BDA has shown significant LDL-C lowering effects, both alone and in combination with ezetimibe, there is reported evidence of some side effects in selected patients. More data will be available after CLEAR Outcome results are available which will further enlighten us about the safety and efficacy of BDA. Further research and trials are needed to significantly conclude the more positive role of BDA in lowering serum LDL-C and preventing cardiovascular events in clinical practice, particularly for patients with CAD or coronary heart disease. 
